# Partial Extensor Hallucis Longus Injury Following Lisfranc Fixation Loosening

**DOI:** 10.7759/cureus.3159

**Published:** 2018-08-18

**Authors:** Peter Adamson, Daniel R Kunzler, Cory F Janney, Vinod Panchbhavi

**Affiliations:** 1 Department of Orthopaedic Surgery and Rehabilitation, University of Texas Medical Branch, Galveston, USA; 2 School of Medicine, University of Texas Medical Branch, Galveston, USA; 3 Department of Orthopedics, Naval Medical Center, San Diego, USA; 4 Department of Orthopedics Surgery and Rehabilitation, University of Texas Medical Branch, Galveston , USA

**Keywords:** lisfranc, extensor hallucis longus, bridge plate

## Abstract

Lisfranc injuries are typically treated in the acute setting with open reduction and internal fixation (ORIF). The type of hardware that provides the best fixation for these injuries has not been definitively determined. Recently, dorsal bridge plating has increased in popularity. We report a case of partial extensor hallucis longus (EHL) injury after dorsal bridge plate fixation of a Lisfranc injury. The patient was successfully treated with hardware removal, tendon debridement, and tubularization. This case highlights a potential complication of dorsal bridge plating in the treatment of Lisfranc injuries.

## Introduction

Tarsometatarsal fracture-dislocation, more commonly known as a Lisfranc injury, can lead to long-term disability and painful post-traumatic arthritis if left untreated [1-4]. These injuries are rare, often misdiagnosed, and can be challenging to manage even for experienced providers [1,4-6]. The current preferred treatment of acute unstable Lisfranc injuries is open reduction and internal fixation (ORIF) [7-8]. Primary arthrodesis is also emerging as a promising surgical alternative, particularly in chronic or purely ligamentous injuries. Currently, the hardware implant that delivers superior outcomes in ORIF is debatable [1,6-7].

Significant damage to the Lisfranc joint is uncommon, with a reported incidence of one per 55,000 individuals, or approximately 0.2% of all orthopaedic traumatic injuries [1,6-7,9]. The majority of injuries result from high-energy forces, though occasionally they originate from a less traumatic mechanism such as a fall with rotational forces on a hyperplantar-flexed foot [10-11].

Timely diagnosis and treatment are the foundation of preventing long-term disability. Regrettably, up to 20% of Lisfranc injuries are either misdiagnosed or undiagnosed [5,7,12]. In chronic injuries, the compromised joint shifts biomechanical forces, leading to increased metatarsal head pressure, deformation of the metatarsal arch, and secondary degenerative joint disease [2,9]. Post-traumatic arthritis is the most common complication [4]. Additional documented sequelae include complex regional pain syndrome, pes planus, neuromas, infections, and vascular injuries [11,13].

Treatment must restore a stable anatomic reduction in a timely manner [5,14]. When deciding the method of fixation, the orthopedist has many options, including Kirschner wires (K-wires), transarticular screws, and dorsal bridge plates. At present, orthopaedic hardware development has surpassed the pace of high-quality research, and no consensus regarding the ideal fixation device for Lisfranc injuries exists [1,4,6-7,9,14]. While early research of dorsal bridge plates is promising, we recently treated a patient suffering from a unique complication. Herein, we report the first extensor hallucis longus (EHL) partial rupture caused by a dorsal bridge plate in the PubMed Central database.

## Case presentation

The Institutional Review Board's approval was waived for this case report in accordance with our institution's policies. A healthy 65-year-old female presented to the orthopaedic foot and ankle clinic three weeks after “twisting her right ankle”. Pain was characterized as diffuse, constant aching, and 7 out of 10. Ibuprofen and Tylenol provided only minimal relief. The foot was generally tender to palpation and range of motion was restricted by pain.

**Figure 1:**
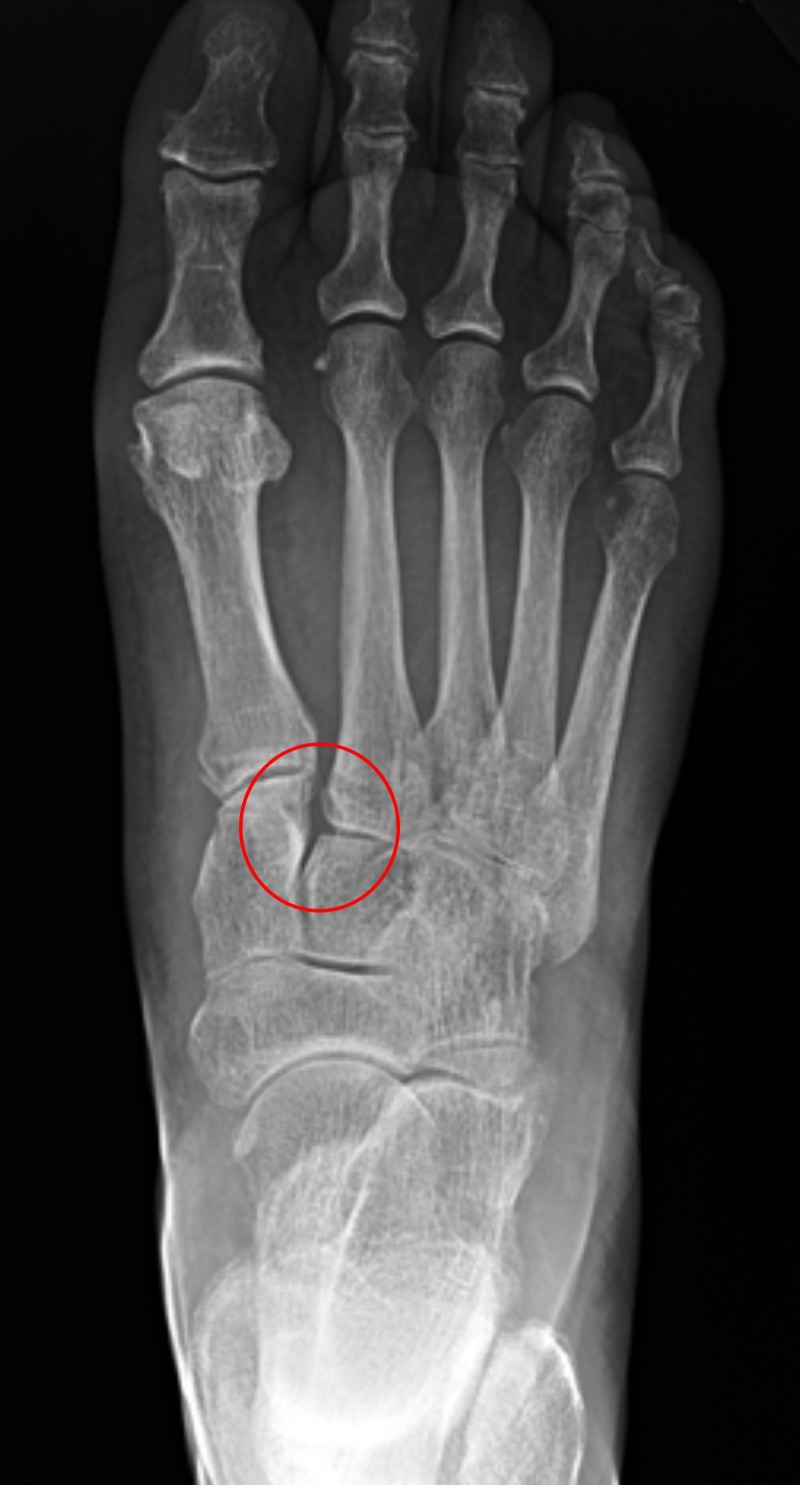
Red circle of the right foot X-ray highlights widening between the first and second ray, which is consistent with Lisfranc injury

On gross examination, moderate edema and plantar ecchymosis were noted. The skin was intact. Vascular and neurological evaluation were normal. Ipsilateral knee and hip evaluation were unremarkable. Her past medical history was non-contributory. Three-view radiographic examination of the right foot revealed widening of the interval between the second and third metatarsals consistent with a Lisfranc injury, as seen in Figure 1.

**Figure 2:**
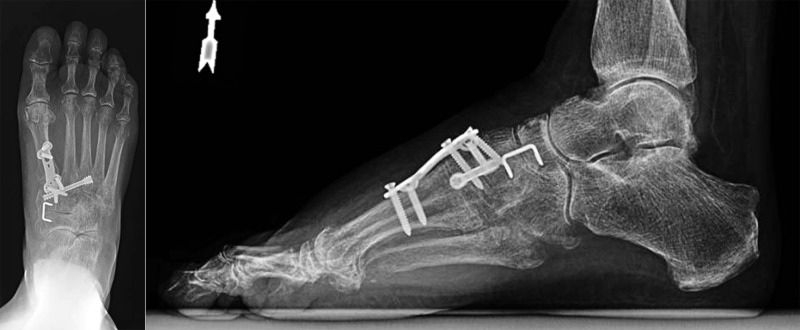
Postoperative lateral radiograph shows fixation with dorsal metatarsophalangeal plate

After a clear explanation of the condition, the patient desired surgical intervention. In the operating room, the Lisfranc tarsometatarsal joint was reduced with a Weber clamp. A K-wire was placed from the base of the third metatarsal into the medial cuneiform. The proper length cannulated, and a compression screw was selected and placed over the wire. The first ray was then assessed and capsular disruptions at the metatarsalcuneiform (MTC) joint and naviculocuneiform (NC) joint were identified. An anchorage metatarsophalangeal plate was placed in a secure fashion over the MTC joint. Next, the NC joint was identified and a staple was used to stabilize the joint. Postoperative films can be seen in Figure 2.

**Figure 3:**
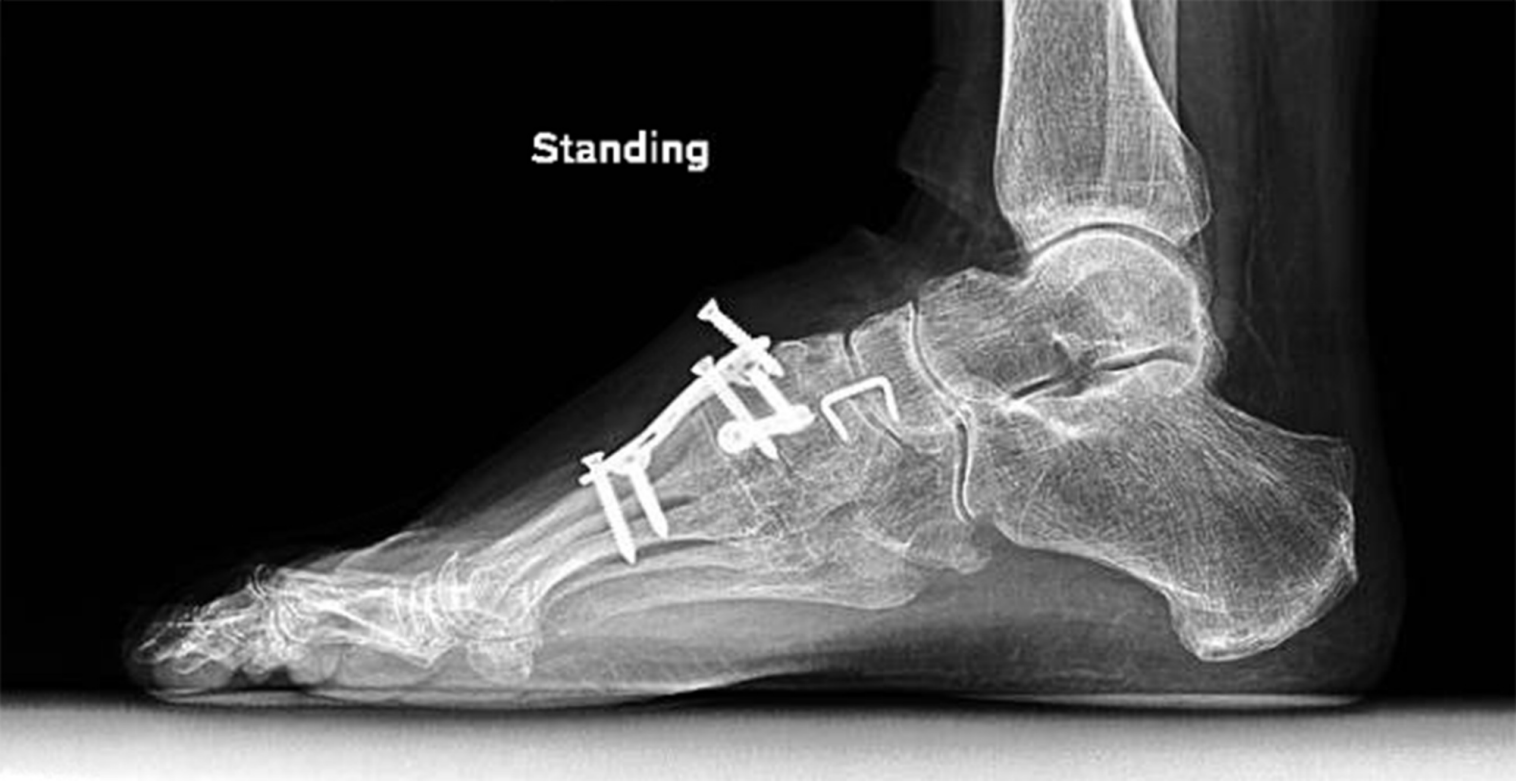
Eight-month follow-up radiograph shows the posterior-most fixation screw backed out of the dorsal metatarsophalangeal plate approximately 9 mm

Postoperative directions included a non-weight bearing for six weeks. She gradually weaned out of a boot and transitioned to a normal shoe with molded insert. She was recovering as expected with a marked improvement in pain until her five-month follow-up appointment. At five months, she began to have short episodes of recurrent pain and lost the ability to dorsiflex her right great toe. At this time, she desired continued conservative management, as she was improving. At eight months, she returned to the clinic with a notable elevation on the dorsum on her right great toe. As seen in Figure 3, radiographs showed the most proximal screw within the dorsal plate had backed out approximately 9 mm.

**Figure 4:**
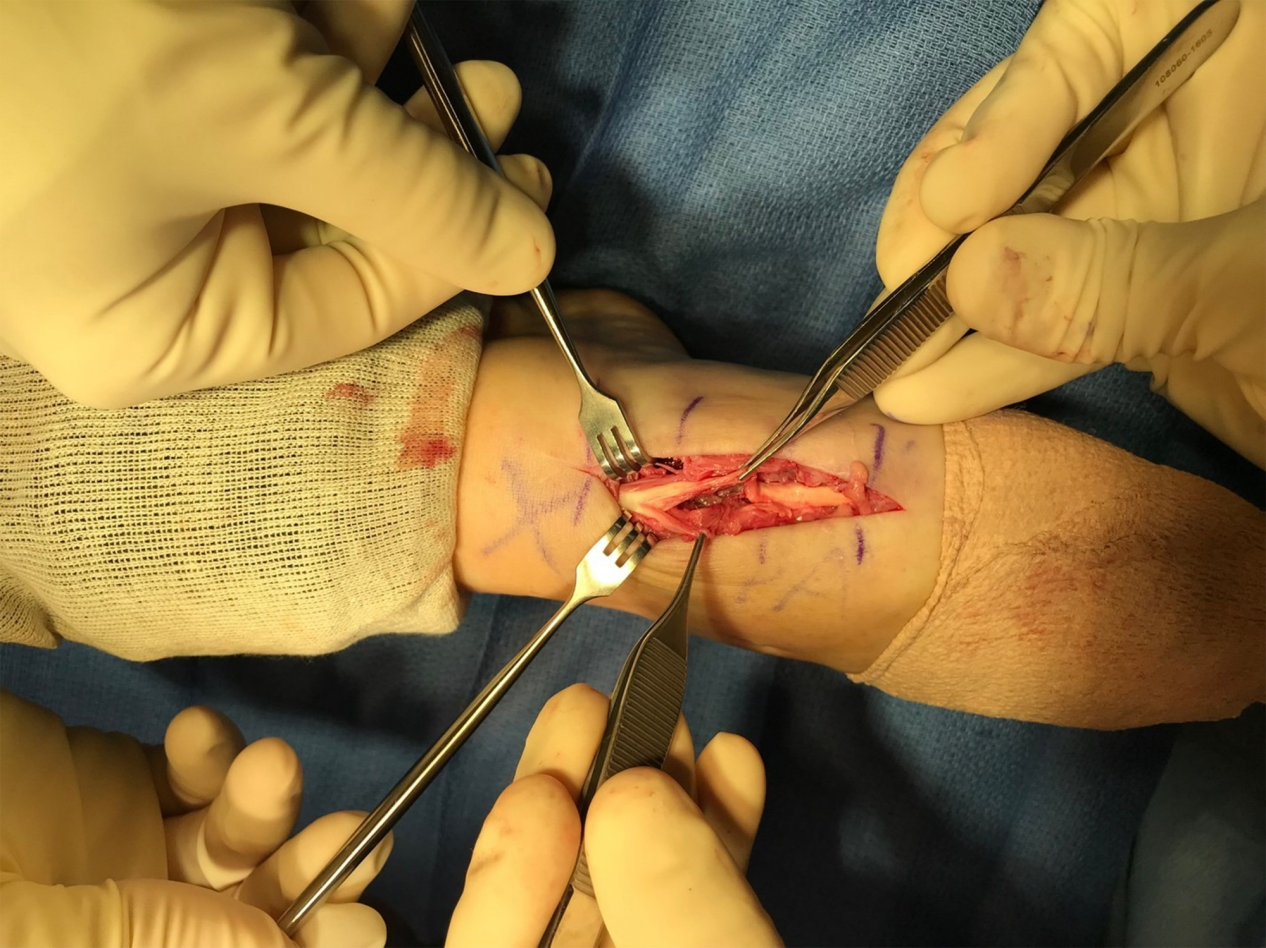
Intraoperative photograph shows a split extensor hallucis longus (EHL)

**Figure 5:**
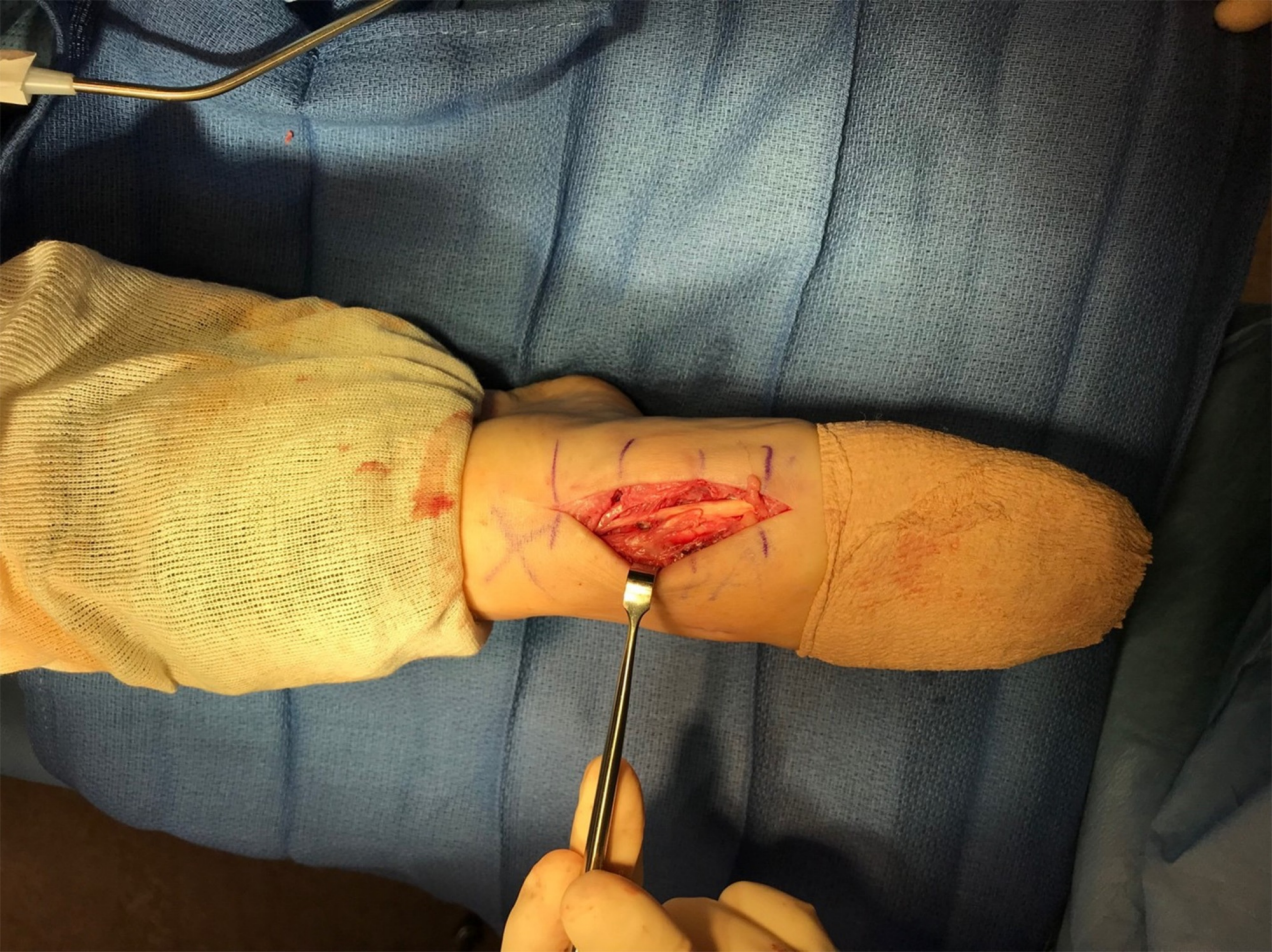
Intraoperative photograph shows repaired extensor hallucis longus (EHL) tendon

Surgery was scheduled, and within two weeks she returned to the operating room. An incision was made through the previous scar, and dissection was carried down to the Lisfranc hardware. Close examination of Figure 4 reveals the etiology of impaired great toe dorsiflexion. Directly overlying the prominent screw, a partial EHL rupture of approximately 50% of the tendon was identified. All hardware was removed, and the EHL tendon was debrided and tubularized, which can be seen in Figure 5.

**Figure 6:**
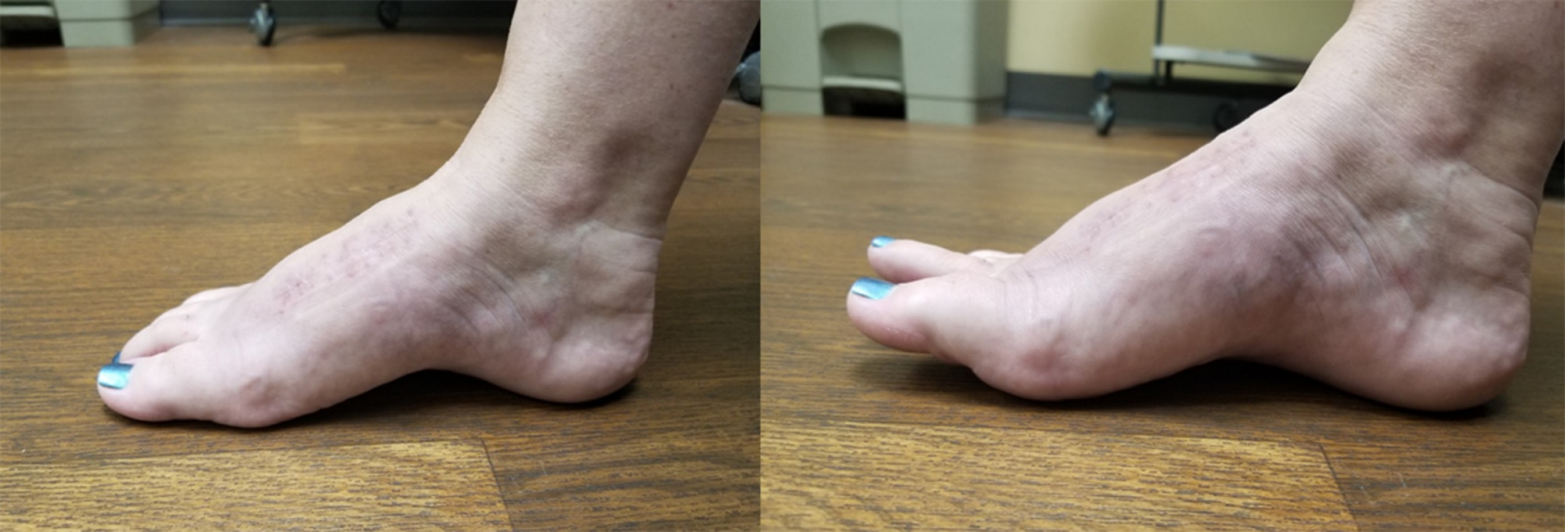
Foot at rest (left) and hallux dorsiflexion (right) is shown after surgical removal of the dorsal metatarsophalangeal plate

The patient was allowed to bear weight immediately following surgery. At two weeks, she transitioned to normal shoes. She had returned to baseline activities, including hiking, by her three-month follow-up appointment. Her range of motion was symmetric to her contralateral side, which can be seen in Figure 6. She had no complaints of pain and was encouraged to follow up as needed.

## Discussion

Traditionally, 2 mm of tarsometatarsal joint displacement is an indication for surgical intervention [2,15]. The interface of the forefoot and midfoot relies on an intimate relationship between the articular surfaces of adjacent bones and support from connective tissue. Even minor degrees of displacement of the tarsometatarsal joint results in significant changes in the joint contact area. For example, dorsolateral displacement of the second metatarsal of 1 mm, 2 mm, and 3 mm results in a reduction in the contact area of 13%, 15%, and 39%, respectively [2,15]. Several studies have demonstrated that clinical outcomes are associated with the degree to which anatomic reduction and stabilization are achieved [1,4,6-7,14].

There is controversy regarding which type of hardware fixation maximizes the benefit-to-risk ratio [1,6-7]. There are advocates for open or closed reduction with K-wires, transarticular screws, and dorsal bridge plates. Fixation with transarticular screws is most commonly used for the first three metatarsal rays, while K-wires are generally preferred for metatarsal rays four and five [3-4,16]. A distinct advantage of K-wires is that no second surgery is needed for hardware removal, as they are typically removed between four to eight weeks postoperatively in the clinic [4]. The drawback of K-wires is that they are inherently weaker, and thus require a longer immobilization period [4,10].

Screws have two main advantages over K-wires. First, they provide rigid fixation, which facilitates early mobility. Second, screws offer more consistent, gentle compression across the joint line, which facilitates healing [2,4,6]. However, screws further damage the articular joint surfaces, which some experts believe contributes to the high rate of post-traumatic arthritis [2-4,6,16]. In the event a screw breaks, further violation of the cortex is inevitable during extraction [2]. Moreover, an additional procedure is typically required, as many orthopedic surgeons remove the screws between eight and 16 weeks postoperatively [4].

Dorsal bridge plates offer several advantages over screws. Plates are able to maintain comparable biomechanical strength without violating the joint surface [1-3,5,16]. In the event of hardware failure, a plate is easier to remove and less likely to cause articular damage in the interim period between failure and surgical removal. Conversely, removal is often necessary, as spanning the tarsometatarsal joint often causes appreciable restriction of the midfoot range of motion [1-2,11]. The requirement to contour each plate also increases operating room time. In addition, our case reveals the previously unknown risk of EHL tendon rupture.

The theoretical advantages of dorsal plates are enticing. A small, prospective study published in 2014 concludes that dorsal plate fixation had superior short- and medium-term outcomes over screw fixation [7]. However, to date, there is a dearth of high-quality, sufficiently powered research regarding the use of dorsal bridging plates in Lisfranc injuries, placing surgeons in an unfortunate predicament. In the early 1990s, the hypothetical advantage of Pi and Forte dorsal radius plates lead to their widespread use. However, these plates quickly fell out of favor due to frequent tendon complications [17]. Our hope is that further research will reveal whether dorsal plates are the gold standard of fixation for the metatarsophalangeal joint, or if we are simply repeating a history of unnecessary iatrogenic tendinopathies.

## Conclusions

While early research of dorsal bridge plates is promising, research regarding their efficacy and side-effect profile is limited. This case documents that EHL rupture is now a possible complication of dorsal bridge plating.
